# From farmers to livestock keepers: a typology of cattle production systems in south-western Burkina Faso

**DOI:** 10.1007/s11250-020-02241-6

**Published:** 2020-03-03

**Authors:** B. Zoma-Traoré, A. Soudré, S. Ouédraogo-Koné, N. Khayatzadeh, L. Probst, J. Sölkner, G. Mészáros, P. A. Burger, A. Traoré, M. Sanou, G. M. S. Ouédraogo, L. Traoré, D. Ouédraogo, B. Yougbaré, M. Wurzinger

**Affiliations:** 1 Division of Livestock Sciences, Department of Sustainable Agricultural Systems, University of Natural Resources and Life Sciences, Vienna, Austria; 2 Institute of Rural Development, Nazi BONI University, Bobo-Dioulasso, Burkina Faso; 3 Department of Life and Earth Sciences, Norbert ZONGO University of Koudougou, Koudougou, Burkina Faso; 4 Institute for Development Research, Department of Sustainable Agricultural Systems, University of Natural Resources and Life Sciences, Vienna, Austria; 5 Institute of Population Genetics, Department of Biomedical Sciences, University of Veterinary Medicine, Vienna, Austria; 6 Department of Animal Production, Environmental and Agricultural Research Institute, Ouagadougou, Burkina Faso

**Keywords:** Farming system, Indigenous cattle, Burkina Faso, Lobi taurine cattle, Zebu cattle, Crossbreds

## Abstract

Cattle production is an essential livelihood strategy in south-western Burkina Faso. Although having a distinct cultural role and known to be resistant against African animal trypanosomosis, the Lobi taurine cattle breed is endangered due to its low market value. As the first step in preservation efforts, our study aimed to develop a typology of production systems at the farm level. We used a structured questionnaire and focus group discussions for collecting data on household characteristics, socioeconomic activities, livestock, and access to services. The sample comprised 169 households in three communities. The analytical strategy included factor analysis of mixed data and hierarchical clustering. We identified four distinct types of cattle production systems: (1) sedentary Lobi farms, (2) sedentary crossbreed farms, (3) semi-transhumant Fulani zebu farms, and (4) transhumant Fulani zebu farms. Significant factors in developing this typology were the farmers’ ethnic group, crop diversity, cattle herd size, cattle herd composition, number of small ruminants, and livestock management strategies. Across all production systems, men were considered being primary decision-makers in cattle production, with women, herders, and children being responsible for specific tasks. All identified production systems are increasingly confronting disease pressure and scarcity of water and land. Future efforts in preservation and breeding will need to respond to these trends in the agroecosystem, integrate risk management measures, and resonate with the specific needs of the different household members involved in cattle rearing.

## Introduction

In Burkina Faso, the cattle production sector contributes between 36 and 40% to total agricultural added value and 26% to agricultural export value (FAO 2018; MAHRH 2011). Two species of cattle, *Bos taurus* and *Bos indicus*, are kept by farmers, agro-pastoralists, and pastoralists. Cattle are a valuable source of food (meat and milk products), provide services (transport and traction), function as a savings and insurance, and play a central role in the culture of different ethnic groups (Jahnke 1982; De la Rocque et al. 2001). The production strategies are based on local cattle breeds and have been described as extensive systems, including mixed crop-livestock, agro-pastoral, and pastoral systems (Kaboré 2012). Members of the Lobi ethnic group, practicing sedentary mixed crop-livestock farming, have traditionally kept Lobi taurine cattle (Coulibaly 1989; Sicot 1993, Mopaté et al. 2014). Lobi taurine cattle are known to be resistant against African animal trypanosomosis (Sow et al. 2005; Dayo 2009; Soudré et al. 2013). However, this breed is unpopular, mainly due to its small size and low market value. Consequently, livestock keepers frequently crossbreed Lobi taurines with larger Fulani zebu. This practice threatens the Lobi as a breed, which has, therefore, been classified as endangered (Sokouri et al. 2009). The Fulani zebu breed originates from the semiarid north of Burkina Faso and is traditionally reared by Fulani pastoralists, who move with their herds to the southern region of the country in search for pastureland and water. Previous studies described the cattle production systems in the region concerning the socioeconomic use of cattle (Coulibaly 1989), different technical management parameters (Sicot 1993), and the degree of integration with crop production (Tano et al. 2001). Mopaté et al. (2014) evaluated the castration of bulls of other breeds as a practice to ensure the conservation of the Lobi breed.

Given the farmers’ preference for breeds with high market value, the productivity of Lobi taurine needs to be improved if the breed is to be preserved. Although Lobi cattle have low productivity in terms of meat and milk, they fulfill a fundamental role in Lobi society and are used in specific cultural events. Therefore, proper management of the breed is relevant to maintain it as an integral part of cultural identity (FAO 2015).

To achieve this, community-based breeding programs (CBBPs), an approach to involve livestock keepers in systematic breeding and management efforts, could be a viable option. CBBPs have been successfully implemented to improve mainly small ruminant production—e.g., of Djallonké sheep in Cote d’Ivoire (Yapi-Gnoaré 2000), Deccani sheep in India (Nimbkar et al. 2002), dairy goats in Mexico (Wurzinger et al. 2013), sheep in Ethiopia (Duguma et al. 2011; Haile et al. 2013; Mirkena et al. 2012), and goats in Iran and Kenya (Mueller 2013; Ojango et al. 2010).

For implementing CBBPs, a thorough understanding of current production systems and farmers’ needs is essential (Sölkner et al. 1998; Kruska et al. 2003; Dossa et al. 2009; Scherf and Tixier-Boichard 2009; FAO 2010; Wurzinger et al. 2011; Robinson et al. 2014). As a first step in preservation efforts, our study aimed, therefore, to develop a typology of production systems at the farm level in south-western Burkina Faso.

## Materials and methods

### Study area and sites

The study was carried out in the south-western region of Burkina Faso, located at latitude of 10°19`00’N and longitude of 3°10`00’W, covering about 16,533 km^2^ (MEF/DREP 2014). The region lies in the mountainous South Sudanese phytogeographical zone, with a rainy season from April to October and a dry season from November to March. The annual precipitation totals between 900 and 1200 mm, with temperatures ranging from 21 to 32 °C (ANAM 2017). Forest and savanna dominate the vegetation (MAHRH/GTZ 2014). About 850,000 people live in the region, and the population growth rate is about 4.5%, including a positive net migration rate of 2% (INSD 2018). The population is composed of different ethnic groups, which are considered being local (Lobi, Dagara, Birifo, Djan, and Pogouli) or immigrants from other regions of Burkina Faso (Mossi, Fulani, and Bobo).

For the research, we focused on the administrative units of Bouroum-Bouroum, Kampti, and Loropeni in the Poni province. The province is typical for the region in terms of pastoral and agricultural system dynamics, as it attracts an influx of migrants from areas with less rainfall and higher chances of drought. In the province, all three types of cattle which are common in Burkina Faso are kept: zebu, taurine, and crossbreds between them.

### Data collection

We collected data using a structured questionnaire and focus group discussions (FGD) from May to September 2018. The sampling population included farmers and pastoralists of three municipalities. For lack of a registry of agricultural producers in the area, we could not apply probability sampling and resorted to a purposive sampling strategy. We collaborated with local extension workers and farmer leaders to identify households that represent the diversity of production systems in the region, and 169 heads of household (all male) agreed to participate. We tested the questionnaire with 15 farmers as a means to improve the final design of the research instrument. We tested the questionnaire with 15 farmers as a means to improve the final design of the research instrument.

The questionnaire comprised household characteristics, socioeconomic activities, livestock data, and access to services such as input supply, credit, and veterinary services. Farmers were asked to score production and management constraints by applying a scale of 1 to 4 (1 = not important/least serious and 4 = very important/most serious).

In a second step, we held separate focus group discussions with Lobi and Fulani respondents to triangulate and illustrate the survey results with qualitative data. Twenty Fulani men and 17 Fulani women attended the first focus group discussion, and 35 Lobi men and 25 Lobi women attended the second focus group discussion. To reduce possible gender effects on the discussion dynamics, men and women were invited to work on the same questions in separate groups. For validation and further discussion, each group then shared the results in a plenary setting. With the participants’ consent, we audio recorded the discussions and documented visual exercises with a digital camera.

All activities were carried out in the local languages preferred by respondents (Dioula, Moore, and Lobiri).

### Data analysis

The qualitative data collected in the FGDs were compiled as written notes for triangulation and interpretation of the survey data.

The survey data were entered into Excel and analyzed using R (v 3.6.1). To explore the data, we used descriptive statistical parameters (mean, standard deviation, minimum and maximum).

**Fig. 1:**
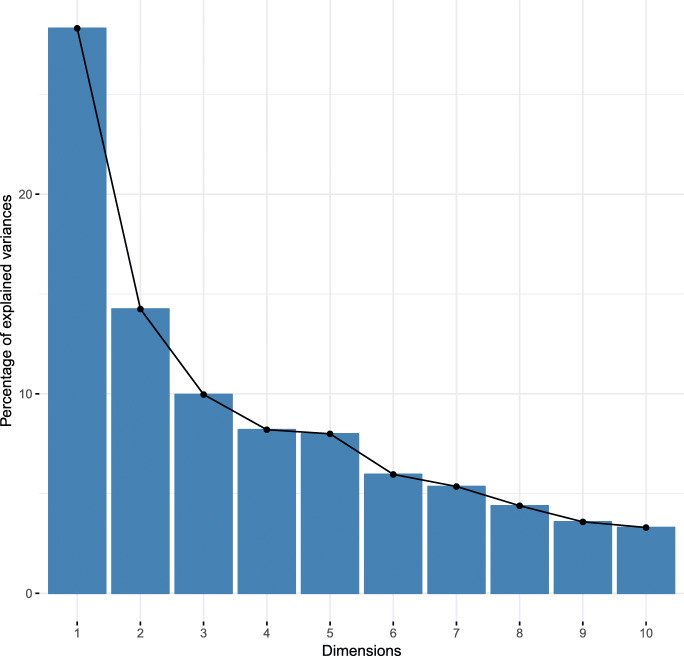
Scree plot illustrating the percentage of variation explained by dimension

To develop a typology of production systems, we applied hierarchical clustering on principal components (HCPC). We reduced the dataset dimensions into non-correlated dimensions, explaining much of the variance of the original dataset, using factor analysis of mixed data (FAMD). FAMD allows conducting a principal component analysis on datasets containing both categorical and continuous variables. Subsequently, we performed a hierarchical cluster analysis (HCA). As input to the FAMD, we used sixteen variables (4 categorical and 12 continuous) (Table 1). We interpreted the scree plot (Fig. 1) to determine the appropriate number of dimensions to be retained for clustering (Joliffe 1986). The hierarchical clustering was performed using Ward’s method, and the gap statistic (Tibshirani et al. 2001) was employed to infer the most appropriate number of clusters. This is done by bootstrap iterations until convergence is reached. The analysis and visualization were performed using the *FactoMineR* and *factoextra* packages in R. The identified clusters were compared using *x*^2^ tests for categorical variables and the nonparametric Kruskal-Wallis test followed by Wilcoxon-tests with Bonferroni-Holm correction for pairwise comparison of continuous variables. Statistical differences were considered significant at *p* < 0.05. The production constraints were ranked using rank means.

**Table 1:** Sample characteristics

*Categorical variables*	*Category*			*n*	*%*
Ethnic group	Fulani			58	34.32
	Lobi			111	65.68
Hiring labor	Yes			91	53.85
	No			78	46.15
Cattle purchase	During the past 12 months			50	29.59
	Not during the past 12 months			119	70.41
Cattle feed supplement	Used during dry season			165	97.63
	Not used			4	2.37
*Continuous variables*	*Description*	*Mean*	*SD*	*Min*	*Max*
Total farmland area	Farm size (ha)	4.7	3.84	0	20
Total cashew area	Cashew farm size (ha)	2.2	4.45	0	30
Crop diversity	Number of vegetable varieties grown	3.3	1.50	0	8
Cattle (excl. oxen)	Number of cattle (excl. oxen) in the herd	53.01	67.47	0	400
Oxen	Number of oxen in the herd	2.47	2.01	0	10
Zebu	Number of zebu in the herd (head of cattle)	32.19	63.67	0	404
Crossbred	Number of crossbred in the herd	14.69	31.74	0	202
Taurine	Number of taurine in the herd	8.6	10.96	0	64
Sheep	Number of sheep	14.91	17.61	0	110
Goat	Number of goats	12.28	11.66	0	50
Vaccinations per year	Number of vaccinations per year	2.39	1.36	0	5
Cattle sold	Number of cattle sold during past 12 months	4.87	7.46	0	47

## Results

### Typology of production systems

The characteristics of the sample are summarized in Table 1.

We included sixteen variables (Table 1) in the FAMD and retained four principal components based on the scree plot (Fig. 1): the scree curve is steep, and the “elbow” is located at four dimensions (cutoff point). These four dimensions describe 62.15% of the total variance (Table 2).

**Table 2:** Results of FAMD: factor loadings

Name of Variables	Components			
	1	2	3	4
Hiring labor	**− 0.664**	− 0.188	0.196	0.361
Ethnic group	**0.877**	− 0.192	0.212	− 0.152
Cattle purchase	0.436	− 0.347	0.036	0.031
Cattle feed supplement	− 0.143	− 0.330	− 0.042	0.491
Total farmland area	**− 0.566**	**0.562**	− 0.011	0.044
Total cashew area	− 0.287	0.369	− 0.467	− 0.107
Crop diversity	**−0.745**	0.306	−0.199	0.144
Cattle (excl. oxen)	**0.757**	0.411	− 0.100	0.422
Oxen	− 0.210	**0.711**	0.073	− 0.033
Zebu	**0.775**	0.211	− 0.038	0.462
Crossbred	0.236	0.446	− 0.232	− 0.133
Taurine	**− 0.569**	0.145	0.293	0.291
Sheep	0.162	0.224	**0.734**	− 0.153
Goat	− 0.048	0.379	**0.694**	− 0.044
Vaccinations per year	**0.628**	0.322	− 0.150	− 0.379
Cattle sold	**0.751**	0.308	− 0.020	0.406
Eigenvalues	4.96	2.18	1.54	1.26
Variance (%)	31.03	13.62	9.64	7.86
Cumulative variance (%)	31.03	44.65	54.29	62.15

N.B. Bold numbers refer to loadings higher than 0.5

The cluster analysis yielded four distinct clusters, which we subsequently compared to develop the typology of production systems. For each cluster, we chose a name that represents its most characteristic features (Table 3).

**Table 3:** Characteristics of different production system in south-western Burkina Faso

	Cluster			
	1	2	3	4
	Sedentary Lobi taurine *n* = 68	Sedentary crossbreed *n* = 42	Semi-transhumant Fulani zebu *n* = 45	Transhumant Fulani zebu *n* = 14
*Ethnic group*				
Fulani (persons)	0	0	44	14
Lobi (persons)	68	42	1	0
*Household attributes*				
Household size (persons: mean/SD)	14.8^a^/8.34	16.2^a^/7.82	10.8^b^/4.98	16^a^/6.09
Age of household head (years: mean/SD)	55.4^a^/11.33	51.4^ab^/11.43	46.2^b^/13.55	49.9^ab^/8.43
*Education of household head*				
Literate (%)	10.29	35.71	11.11	7.14
Illiterate (%)	89.71	64.29	88.89	92.86
*Main purpose of cattle production*				
Cattle for saving/insurance (%)	10.30	47.62	0.89	14.29
Cattle as draught animal (%)	58.82	38.09	00	00
Cattle for sacrifices, dowry and others social events (%)	30.88	14.29	00	00
Cattle as main source of livelihood (%)	0	0	91.11	85.71
*Livestock ownership and management*				
Cattle excl. Oxen (number of animals: mean/SD)	18.1^a^/12.96	49.2^b^/55.10	58.8^c^/36.89	215.4^d^/93.94
Oxen (number of animals: mean/SD)	2.6^a^/2.13	3.2^a^/2.41	1.5^b^/1.12	2.3^ab^/1.07
Total cattle (number of animals: mean/SD)	20.7^a^/13.54	52.4^b^/55.51	60.3^b^/37.24	217.7^c^/94.23
Taurine (number of animals: mean/SD)	17.3^a^/11.90	5.3^b^/4.79	0.9^c^/3.27	0.9^c^/2.67
Crossbred (number of animals: mean/SD)	1.1^a^/2.51	33.6^b^/48.79	14.1^c^/21.15	26.1^bc^/41.18
Zebu (number of animals: mean/SD)	2.3^a^/4.03	13.6^b^/25.49	45.3^c^/40.49	190.7^d^/106.15
Hired labor (%)				
Yes	8.82^a^	73.81^b^	91.11^c^	92.86^bc^
No	91.18	26.19	8.89	7.14
Cattle bought (%)				
Yes	39.71^a^	50^a^	4.44^b^	00^b^
No	60.29	50	95.56	100
Cattle sold (number of animals: mean/SD)	1.3^a^/1.4	3.1^b^/4.26	6.1^c^/4.91	23.5^d^/10.75
*Small ruminants*				
Sheep (number of animals: mean/SD)	14.6^a^/15.65	6.9^b^/9.21	23.2^a^/23.67	13.6^ab^/11.63
Goat (number of animals: mean/SD)	14.3^a^/11.94	8.7^b^/9.52	12.9^ab^/11.88	10.9^ab^/13.83
*Agriculture*				
Crop farm size (hectares: mean/SD)	6.3^a^/3.82	6.4^a^/3.84	1.6^b^/1.25	2^b^/1.21
Cashew cropping area (hectares: mean/SD)	1.6^a^/2.13	6.3^b^/6.99	0.02^c^/0.15	0.07^c^/0.27

^abc^Means within rows that do not have a common superscript are significantly different at *p* < 0.05 level

The first type of production system—which we refer to as “sedentary Lobi taurine farms”—comprised 40.24% of the interviewees, all of whom were Lobi. This system was characterized by a low number of cattle (an average of 20.7 heads) with the vast majority being taurine cattle (83.57%) for draught, saving and insurance, and social functions such as funerals, sacrifices, and dowry (Table 3). In this system, the cattle were herded by children in the rainy season, and free grazing was practiced after the crop harvest and during the dry season. The frequency of treatment against trypanosomosis was low due to natural resistance in taurine cattle. Farmers rarely sold their cattle, and when they did, it was only in case of urgent financial needs.

**Fig. 2:**
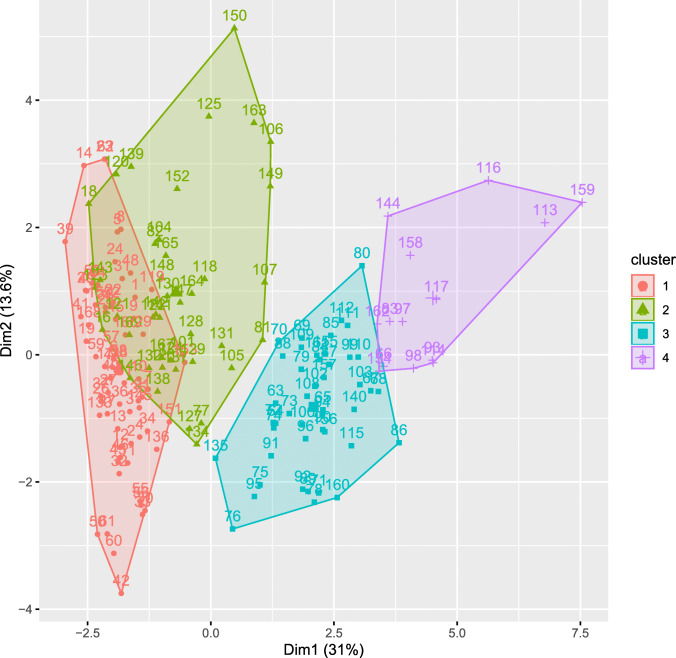
Cluster plot showing the four clusters (outcome of the hierarchical cluster analysis) in the FAMD component 1 and 2 plane

The second system—which we refer to as “sedentary crossbreed farms”—was also focused on crop production, but cattle played a more central role as a mechanism for savings and insurance, as draught animals, and as providers of manure for crop production. Farmers pursuing this strategy raised at least two types of cattle, with the majority being crossbred (64.12%), mainly used as draught animals, according to farmers (FGD). Crossbred animals were also considered more resistant to trypanosomosis than zebu and more profitable than taurine. The average number of cattle per household was about 52 heads (Table 3). The farmers relied on paid workers for herding the animals throughout the year. Similar to the “sedentary Lobi taurine farms” strategy, cattle were not sold regularly (Fig. 2).

In the third production system—which we refer to as “semi-transhumant Fulani zebu farms”—interviewees focused on milk and meat production for the market, with limited crop production of sorghum or millet for home consumption. Contrary to the two first production systems, the vast majority of producers were Fulani (97.78%). Considering the crucial role of livestock for their livelihoods and also the higher number of zebu in this production system (75.12%), the respondents indicated higher costs for fencing, feed, and veterinary services. Furthermore, this system, with an average of about six cattle sold a year, was more cattle market oriented than clusters 1 and 2. The average number of cattle was 60.3 heads per herd, and producers were moving within a local territory during the dry season.

The fourth production system—which we refer to as “transhumant Fulani zebu farms”—resembles cluster three in many features, including the ethnic group, the high number of zebu cattle, and the management system with a high amount of hired labor. The higher average number of 217.7 heads per head conferred the owner a high place in the society and security of vicissitudes of life. However, the high number of cattle forced them to migrate beyond the national boundaries during the dry season regularly. Moreover, they kept their cattle far from settlements during the rainy season to prevent herds from causing damage to farmers’ fields. This system was the most cattle market oriented, with an average of 23.5 heads sold a year.

Across all four systems, cattle management was a family task, and men were widely considered having the primary responsibility for the cattle, supported by women and children. To different degrees, all family members were involved in feeding, watering, vaccination, and construction and cleaning of feedlots. Decisions regarding the purchase of cattle, feed supplements, and veterinary services were mostly the preserve of men. The men were also responsible for preventing animal theft, searching for lost animals, and for solving conflicts with other livestock keepers and farmers. Women were mostly in charge of calves, sick animals, small ruminants, watering animals, and milking. Breeding was not mentioned as a relevant task (FGD results).

The systems differed, however, regarding the distribution of labor. In “sedentary Lobi taurine farms,” family members were the main source of labor. In the “sedentary crossbreed farms” cluster, Fulani laborers managed the cattle. Farmers hired the laborers and covered the costs related to animal treatment and feeding. In the FGDs, women participants of these groups emphasized that they do not consider the distribution of labor and income fair—their contribution to livestock production, including the provision of water in the dry season, was an addition to sustaining the family, while the control over cattle revenues remained with the men.

In the “semi-transhumant Fulani zebu farms” system, cattle management was again a shared family task: herding was carried out by the owners and their children—particularly in Fulani households, in which all boys were herding after school. In this system, women were responsible for milking and milk processing as well as selling milk products. They also produced soap and butter for domestic use and sale. In contrast to women from sedentary farms, women participants in the FGDs of this group were not concerned by the question of sharing income fairly. However, while their husbands were satisfied with the division of labor, women in this group called for more support and appreciation by men.

In cluster four, “transhumant Fulani zebu farms,” the respondents were not directly involved in cattle management as cattle were kept far from the homestead. Laborers managed the cattle, and the owners visited the herds for follow-up only. In this system, women were not regularly involved in cattle production.

### Constraints on cattle production

We summarize the identified constraints on cattle production in Table 4. In all production systems, farmers considered the lack of drinking water for animals, the lack of feed (pasture), and the pressure of diseases and parasites being the primary challenges. Further constraints were the high costs of veterinary drugs and the high mortality of young animals. In general, the number of constraints mentioned increased with the herd size of farmers. The individual perceptions were corroborated in the FGDs, and the participants across all systems considered migration into the area, transhumance, and the growing population as main trends affecting agricultural production. Although water scarcity was frequently mentioned, this was attributed to the high demand rather than a changing climate. Owners of “sedentary Lobi taurine farms” and “sedentary crossbreed farms” suggested that they needed more knowledge to improve cattle management. Owners of “semi-transhumant Fulani zebu farms” and “transhumant Fulani zebu farms” found problems of damage on farmland and conflicts with farmers most constraining on their cattle production.

**Table 4:** *Primary constraints in cattle production in south-western Burkina Faso (mean scores)*

Constraint	Sedentary Lobi taurine farms	Sedentary crossbreed farms	Semi-transhumant Fulani zebu farms	Transhumant Fulani zebu farms
Drinking water	3.33	3.42	3.72	3.89
Lack of pasture	2.9	3.24	3.58	3.75
Diseases and parasites	2.46	3.41	3.52	3.72
Damage on farmland	1.77	2.56	3.02	3.56
Conflict between farmers and breeders	1.3	2.43	2.8	3.43
Young animal mortality	2.05	2.16	2.84	3.21
Feed shortage	1.86	1.97	2.38	2.41
Theft or predators	2.37	2.13	2.02	2.55
High veterinary costs	1.97	2.27	2.38	2.5
Insufficient technical knowledge	2.34	2.22	1.94	1.45
High input costs	1.79	1.91	2.32	2.96
Marketing problems	1.42	1.88	2.04	2.27
Housing problems	2.06	2.03	2.04	2.07
Access to credits	1.38	2.16	1.98	1.5
Access to extension service	1.57	1.73	1.8	1.67

The study participants discussed adaptation pathways in the FGDs. Respondents whose primary occupation was livestock production considered a reduction of livestock density and an improved social organization of different agricultural activities in the region being the main adaptation pathways. Sedentary farmers, however, proposed to focus on the intensification of agricultural production.

## Discussion

### Household characteristics

Age and literacy of household heads across the different production systems found by this study are in accordance with earlier work in the region (Soro et al. 2015; Mopaté et al. 2014). Any effort to fostering breeding programs in the region will need to take into account that the farming population is aging and mostly illiterate. Integrating their knowledge and experiences will be crucial to initiate any learning process for change. Earlier research has documented that literacy is a crucial factor in agricultural innovation (Adeleye et al. 2016).

Also, effective breeding efforts will have to resonate with the preferences and needs of a diversity of persons who have a role in cattle rearing: men as official decision-makers, but also women regarding milk production and processing, as well as hired herders.

### Crop production

An increasing number of production risks confront smallholder agriculture in Sub-Saharan Africa, and agroecosystem diversification has been identified as a main buffer strategy (Altieri et al. 2015; Hänke and Barkmann 2017; Gbegbelegbe et al. 2018). In our study, farm size, market prices, and climate change were drivers for diversifying production systems—which is in agreement with earlier research in the region showing that farmers who own larger plots diversify into profitable cash crops (Ouédraogo et al. 2010; Audouin 2014). Livestock keepers, who are often landless, tend to rent small parcels of land to build a homestead, grow some staple crops, and build a kraal for small ruminants and dairy cattle (see Sanon et al. 2014). Considering that producers have different risk profiles and are generally risk-averse (Wiggins 2016), new breeding programs in the region should explicitly integrate risk management to increase the likelihood of participation.

### Livestock production system

In earlier studies, production strategies and specific breeds were typically described in association with ethnic groups such as Lobi and Fulani (e.g., Mopaté et al. 2014; Soro et al. 2015; Dossa and Vanvanhossou 2016). We found, however, that the categorization based on ethnic groups has become less meaningful for tailoring interventions: the herd sizes of Lobi “sedentary crossbreed farms” were similar to those on Fulani “semi-transhumant Fulani zebu farms.” Moreover, breed preferences have become less clear-cut: some sedentary farmers keep crossbred cattle for improved traction fitness and higher market value, and some semi-transhumant farmers recognized its superior resistance against diseases compared to pure Zebu cattle (see also: Mopaté et al. 2014; Sanon et al. 2014). In the literature, this dynamic is mainly attributed to a change in climate and increasing human migration: stocking herds as a savings strategy following favorable agricultural seasons has led to an increase in the number of zebu cattle in south-western Burkina Faso (INSD 2014). Moreover, larger herds are more likely to be managed using transhumant strategies, as also found by Kaimba et al. (2011). Farmers with larger herds tend to employ herders, which may bring along cattle that are consequently crossbred with the herd owners’ animals (Mopaté et al. 2014; Dossa and Vanvanhossou 2016). Finally, the fact that breeding was not considered a task to be managed in livestock production implies that interventions would first have to establish the benefits and costs of systematic breeding jointly with men, women and laborers.

### Constraints on cattle production

Our study adds to the evidence that lack of drinking water for animals, the lack of feed (pasture), and the pressure of diseases and parasites are the primary challenges for livestock production in the region—as reported in previous studies (Soro et al. 2015; Koutou et al. 2017). Dossa and Vanvanhossou (2016) explained the decline of the Somba cattle population in Benin with the high mortality due to diseases, and feed and water shortage. According to Soudré et al. (2013) and Soro et al. (2015), trypanosomosis is a disease strongly reducing productivity in the region. Farmers, however, reported that over the past several years, the effect of foot and mouth disease has been even more adverse (Soudré et al. 2013). A likely contagion mechanism is transhumance during the dry season. In general, the identified constraints show the interlinkages of regional socio-ecological systems: the shortage of water has been explained by increased crop and cattle production in the region, as well as changing rainfall patterns. From 2006 to 2016, the farmed land in the region increased by 110% and cattle production increased by 31% (INSD 2016, 2018). The high international demand for cashew nut has contributed to the intensification of agriculture (Audouin 2014). Finally, the growing population in the region has further reduced the land available as pasture (Koutou et al. 2017). This shortage has likely increased conflicts between farmers and cattle keepers, who are competing for the same resources (Hellemans and Compere 1990; Vall et al. 2006; Gonin and Tallet 2012a, b.). Our conclusion is that breeding programs need to adopt a systemic perspective to integrate meaningfully with current trends in the agroecosystem.

### Methodology

The results of this study should be interpreted taking into account the limitations of survey research. First, we must assume that the sample is not fully representative of the farmer population in the area. Second, the perspective of the household head may not fully reflect the realities experienced by other household members. Third, in standardized questioning, respondents make assumptions about the meaning of questions and potential answers (Strack and Schwarz 1992). These assumptions may lead to biases (e.g., social desirability).

We aimed to mitigate these limitations by (1), using a purposive sampling strategy to include the diversity of production systems, while not concluding on the quantitative ratio of the different types; by (2), complementing the household survey with FGDs to integrate the perspective of women and to validate the survey findings.

## Conclusion

The purpose of this study was to understand cattle production systems in south-western Burkina Faso, given the importance of the sector for livelihoods and the endangerment of the local Lobi taurine breed. We identified four distinct types of production systems: (1) sedentary Lobi farms, (2) sedentary crossbreed farms, (3) semi-transhumant Fulani zebu farms, and (4) transhumant Fulani zebu farms. While Lobi taurine and crossbred animals continue to fulfill different livelihood and cultural roles, Lobi farmers have started to invest in cattle rearing as a complementary livelihood strategy. Fulani pastoralists have started to engage in crop production and continuous local marketing of animal products. Accordingly, traditional categories of “Lobi farmer” and “Fulani livestock keeper” do not fully reflect the reality of the sector—future preservation and breeding efforts must take this transition into account. Moreover, all identified production systems are increasingly confronting disease pressure and scarcity of water and land. Cattle breeding programs will need to respond to these trends in the agroecosystem, integrate risk management measures, and resonate with the specific needs of the different household members involved in cattle rearing.
